# Personal and Lifestyle Determinants of HIV Transmission Risk in Spanish University Students

**DOI:** 10.3390/ijerph17228332

**Published:** 2020-11-11

**Authors:** Cristian Alcocer-Bruno, Rosario Ferrer-Cascales, Nicolás Ruiz-Robledillo, Miriam Sánchez-SanSegundo, Ana Zaragoza-Martí

**Affiliations:** 1 Department of Health Psychology, Faculty of Health Science, University of Alicante, 03690 Alicante, Spain; cristian.albru@ua.es (C.A.-B.); miriam.sanchez@ua.es (M.S.-S.); 2 Department of Nursing, Faculty of Health Science, University of Alicante, 03690 Alicante, Spain; ana.zaragoza@ua.es

**Keywords:** HIV, risk, lifestyles, university, students

## Abstract

The increase in human immunodeficiency virus (HIV) transmission cases poses a serious public health concern. Although several previous studies have been conducted with the aim of identifying the risk factors for HIV transmission, the number of cases has been increasing, especially in youth. The present study is aimed at the identification of personal and lifestyle determinants of HIV transmission risk in a sample of 335 Spanish university students selected by convenience sampling from a public university located in Alicante (Spain). Sociodemographic factors, lifestyles, and variables of HIV risk of transmission were evaluated. Group differences on risk of HIV transmission were evaluated between participants depending on their sociodemographic characteristics (age, sex, relationship status, employment status, economic status, and sexual orientation) and lifestyle (diet, physical exercise, smoking, alcohol consumption, and stress). Linear regression models were conducted in order to identify those personal and lifestyle variables related to HIV transmission risk. The obtained results indicate that, generally, being older, in a relationship, and employed were factors related to a high risk of HIV transmission. Regarding lifestyle, poor diet, lower intensity of physical exercise, higher alcohol intake, and smoking were fundamentally associated with a higher risk of HIV transmission, through lower use of condoms and higher frequency of risky sexual behaviors. Hence, participants who develop an unhealthy lifestyle exhibit twice the probability of being at a high risk of HIV transmission, especially regarding these previously indicated behaviors. The present study points out the relevance of sociodemographic characteristics and lifestyles of university students in their proneness to developing risky behaviors for HIV infection. Future studies should be developed with larger, randomized, and more representative samples, in order to obtain significant information for the development of effective preventive strategies oriented toward the increase in the adherence to healthy lifestyles and HIV prevention.

## 1. Introduction

According to data reported by the World Health Organization (WHO), there were 37.9 million people living with the human immunodeficiency virus (HIV) worldwide at the end of 2019 [1]. Africa is the continent with the largest number of people infected with HIV, representing almost 70% of the total population (25.7 million), followed by Asia (10.02%), the Americas (9.2%), and Europe (6.6%) [1]. In Spain, it has been estimated that, at present, approximately 150,000 people are infected with HIV, with 3100 new cases being registered in 2018 and young people being the most at-risk group [2]. In fact, it has been established that 50% of the new diagnosed cases of HIV in the world occur in people aged between 14 and 24 years [3].

Several mechanisms have been proposed to understand the higher risk of transmission of sexually transmitted diseases (STDs) and HIV in youth [4]. In this regard, higher sexual experimentation, a greater probability of spontaneous sexual encounters, and less knowledge about HIV and the risk factors of sexual partners have been identified as possible explanations for the higher prevalence of STD diagnoses in this population [4]. Hence, having sexual intercourse at an early age has been related to a greater number of sexual partners during the life cycle and less condom use, which increases the risk of STD transmission and acquisition [5,6]. Other studies have demonstrated that the risk of HIV transmission increases significantly among adolescents and university students [7], due to the fact that young people tend not to use a condom and have sexual relations with a greater number of partners than adults [7].

Despite the fact that young people consider condom use to be the most reliable, safe, and comfortable method for STD prevention, their use is influenced by several factors, with age and gender being the most important variables [6]. In this regard, it has been demonstrated that some young people consider the use of condoms a stigmatizing behavior, not feeling confident and comfortable in their employment [3]; for example, the use of a condom may entail that they think their partner is diseased. Further, it could be interpreted as a sign of disease [3]. However, some gender differences should be addressed in this regard. Most young people develop the belief that offering condoms during a sexual relationship endows them with a condition of promiscuity, especially in the case of females [6]. Further, it has been demonstrated that young people think that the use of the prophylactic generates a lack of confidence in the couple, as requesting their use in sexual intercourse could denote mistrust or the fact that one of the members of the couple suffers from an STD, generating several disturbances in the couple [3]. In addition, the use of condoms is conditioned by the person themselves as to whether they consider their sexual partner to be “clean” or not, or whether they are a stable sexual partner. Hence, men in stable romantic relationships, based on the trust of their partner, consider it unnecessary to use a condom [6]. Moreover, females show less assertiveness when requesting and using condoms in sexual relationships [4].

Beyond gender, sexual orientation has been demonstrated to significantly influence the adoption of risky sexual behaviors. Regarding gay or bisexual people, men who have sex with men (MSM) have been demonstrated to be at particularly higher risk of exposure and acquisition of HIV than men who have sex with men and women [8,9,10,11]. This risk is fundamentally based on the sexual risk practices that they carry out and the perception of the lower importance of condom use, minimizing the risk of transmission [12,13].

When the other personal characteristics of individuals have been taking into account, contextual variables, such as socioeconomic or employment status, emerge as significant influential variables for the development of HIV risk behaviors [14,15]. In particular, economic vulnerability (i.e., low income and unemployment) has been associated with an increased risk of HIV transmission. Hence, previous research has pointed out that women who were unemployed suffered higher levels of negative mood and refused the use of condom in sexual intercourse [16]. This fact reinforces the idea that not being employed is strongly associated with condom misuse and, therefore, increases the risk of STD acquisition.

In addition to personal and contextual characteristics, lifestyle plays a significant role in the development of risky sexual behaviors. Previous studies have established a significant association between alcohol consumption and higher risks of transmission of HIV and other STDs [17,18,19], especially in women [4,20]. Similar results have been found in specific populations, such as MSM, who reduce the use of condoms during anal penetration when they are under the influence of alcohol [21]. Furthermore, alcohol consumption has been associated with an increase in the number of sexual partners and the frequency of sporadic sex in this population [22].

Regarding tobacco consumption, smoking has been traditionally characterized as a predisposing factor for acquisition of HIV and other STDs, as indicated by different studies [23,24,25,26,27]. Generally, it has been found that smokers subsequently acquired HIV to a greater extent than non-smokers, in addition to engaging in more risky sexual practices [28]. All of these results of previous research are very relevant, as alcohol consumption and smoking are risky behaviors for health, which are highly prevalent during adolescence and youth [29,30,31].

Although the association between alcohol and tobacco consumption with sexual risk behaviors has been well-established in the previous literature, little is known about the associations with other components of lifestyle, such as diet and physical exercise. Regarding diet, food insecurity has been related to increased sexual risk, through transactional sex and inability to negotiate safer sex in women living with HIV [32], a higher number of male sex partners in the case of women [33], and more unprotected sex acts in men [33]. These results have been replicated in an adolescent population, where food insecurity is a significant predictor of unwanted sexual contact [34]. However, all of this research was related to food insecurity and, taking into account that food insecurity could be more a proxy of a lower socioeconomic status than an independent construct [35], new studies evaluating the relationship between specific diet characteristics and risky sexual behaviors are needed. It has been established that healthy habits usually tend to occur together, and it is highly probable that individuals who develop a higher adherence to a healthy diet exercise more and become less involved in risky behaviors, such as sexual ones [36]. Further, as in the case of physical exercise, a healthy diet has been associated with adequate cognitive functioning [37], and cognition has been related to HIV risky behaviors [38].

For physical exercise, as in the case of diet, the information in this regard is limited and, in some cases, contradictory. Although some studies have found a significant association between higher physical activity and lower sexual risk behaviors [39], others found the opposite relationship; that is, they found an association between higher physical activity and more risky sexual behaviors [40]. Furthermore, other studies did not find any relationship [41]. As it has been previously indicated, in the same way that diet may lead to positive outcomes, physical exercise could entail better cognitive functioning and, hence, lower involvement in sexual risk behaviors [38].

Given the low skills of sexual negotiation, together with the abusive use of alcohol, tobacco, and other drugs and early sexual initiation, multiple sexual partners, and the practice of unprotected sex due to the abandonment of condom use, which may be highly prevalent in youth and entail serious risk factors for HIV transmission. It is very important to identify personal and lifestyle dimensions which could be related to them, in order to establish a profile of youth at risk. As it has been established that habits acquired during youth are related to healthy behaviors in the future, the acquisition of sexual health-promoting behaviors in this population is essential for the prevention of STDs. However, it is necessary to evaluate those personal factors and specific characteristics of the lifestyles of young people, which are related to different risk behaviors of HIV transmission. In this sense, a recent review has indicated that information about HIV, attitudes, and self-efficacy in the use of condoms and safe sexual behaviors are the most important dimensions to be evaluated when the risk of HIV needs to be identified [42]. Hence, in order to advance in the comprehension of the effects of these personal and lifestyle variables in the development of risk of HIV transmission in this population, the present study was aimed to the evaluation of the relationships between these factors in a sample of Spanish university students.

## 2. Materials and Methods

### 2.1. Procedure

The present study was a part of a large-scale cross-sectional study on the relationships between lifestyle, well-being, and risky sexual behaviors in Spanish university students. The participants included 335 young people living in Spain who were selected by convenience sampling from a public university located in Alicante (Spain). This university is located in Alicante, a metropolitan area with more than 750,000 inhabitants. It is a public university in which more than 25,000 students are enrolled every year. Public universities in Spain differ from private ones mainly by funding and student access. Public universities are funded by the state government, which entails that students pay a reduced tuition price in comparison to private universities. Moreover, the access requirement for a public university in Spain is the cut-off mark, established according to the number of students who want to enroll in that career. In private ones, on the other hand, usually, it is enough to pass the university entrance exams.

Student enrollment records of several faculties in which authors of the study give lessons were consulted, and then, they attended personally several classes from these faculties to invite students to participate in the study. Before participation in the study, the members of the research team gave full information about the characteristics and aims of the project in person in class, making clear that the participation was completely voluntary and did not comprise part of their course of learning. Furthermore, it was also explained that the participation would have no effect on their course grades. Only individuals who agreed to participate and signed the informed consent were included in the study and answered the indicated questionnaires anonymously in an online format during class time the same day. The confidentiality and anonymity of the obtained results was assured to participants throughout the whole study. Hence, to protect the confidentiality and anonymity of the data, codes were assigned to identify the participants. Furthermore, the research was conducted following the guidelines of the Declaration of Helsinki and the European Union Good Clinical Practice Standards, and the study was approved by the Ethical Committee of Alicante Institute for Health and Biomedical Research (PI2019-083). Inclusion criteria were: (1) presence in the classroom on the day of the survey; (2) ability to read and complete the questionnaires themselves; and (3) having signed the informed consent to participate in the study. Participants were retained in the final sample only if they responded to all the questions involving the dependent variables evaluating risk of HIV infection and lifestyle. Of the initial sample of 365 potential participants, 18 (4.93%) refused to participate in the study, and 12 (3.29%) did not answer all of the questions in the employed questionnaires. Data were collected by a research assistant during the fourth trimester of the 2019–2020 academic year and sessions lasted approximately 45 min. Personal and sociodemographic characteristics of the sample are summarized in Table 1.

### 2.2. Measures

#### 2.2.1. HIV Risk

For the analysis of HIV risk, the Spanish version of the AIDS Prevention Questionnaire (CPS) [42] was employed. This evaluation instrument contains 44 items with different types of scale responses: 14 dichotomous items, 2 multiple-choice items, 24 Likert-type items, and 4 continuous 0–100 items. This questionnaire is one of the first instruments to evaluate HIV prevention from a comprehensive and multidimensional perspective. Hence, it includes 5 main sub-scales: Knowledge about HIV (K-HIV), which evaluates the subject’s perceived information and real knowledge about risky practices, preventive measures, HIV testing, and the impact of HIV in people living with HIV; Attitudes and Perceived Self-Efficacy (SEA), oriented to the assessment of abilities and skills to use condoms; Condom Use Intention (CUSEI), which evaluates the behavioral intention to use condom in several situations and scenarios; Safe Sexual Behavior (SAS-B), which contains questions regarding the frequency of condom use in different situations and contexts; and Stigma and Discrimination towards people living with HIV (SD-HIV), which evaluates solidarity behaviors and empathy towards people living with HIV. Lower scores are indicative of higher risk and difficulties in each domain. For the present study, only dimensions directly related to HIV risk (K-HIV, SEA, CUSEI, and SAS-B) were employed. This questionnaire was validated with Spanish young people and demonstrated a good reliability index, with all of the sub-scales ranging from 0.67 to 0.74 in the Cronbach Alpha.

#### 2.2.2. Lifestyles

The Simple Lifestyle Indicator Questionnaire (SLIQ) was used to evaluate lifestyles [43]. This questionnaire evaluates five dimensions: diet (3 questions to evaluate frequency of vegetable, fruit, and cereal consumption during the past year), physical activity (3 questions to evaluate the practice and the frequency of practice in an average week of light, moderate, and vigorous physical exercise), alcohol consumption (3 questions to evaluate the intake of wine, beer, and spirit drinks in an average week), smoking (2 questions to asses if the individual is a non-smoker or current or former smoker), and stress (1 question to evaluate the level of stress in the everyday life of the individual). The raw score obtained in each dimension could be employed to categorize participants in three groups for each subscale. In the case of diet, individuals could be categorized in three levels based on their adherence of a healthy diet (poor, moderate, or good diet). For physical exercise, the categories are configured based on the practice of each type of exercise (light, moderate, or vigorous). Categories in alcohol consumption are formed by the number of drinks consumed by participants (low, moderate, or high alcohol consumption). In the case of smoking, individuals are grouped based on three options: non-smoker, former smoker, or current smoker. Finally, for stress dimension, participants are grouped based on the perceived intensity stress by low, moderate, or high stress perception. With the category scores in each sub-scale, a total score about adherence to a healthy lifestyle can be calculated. These overall scores range from 0 (unhealthy lifestyle) to 10 (healthy lifestyle), and, based on a similar established criterion, participants can be categorized based on the total score (low, moderate or high adherence to a healthy lifestyle). This instrument was validated with individuals with hypertension and exhibited adequate psychometric properties, with test–retest reliability ranging from 0.63 to 0.97.

### 2.3. Data Analysis

T-tests were performed in order to assess differences between groups, based on personal and sociodemographic variables, in each dimension of the HIV risk questionnaire. Cohen’s d was calculated, in order to evaluate the size effect of these differences. Multivariate analysis of variance (MANOVA) was employed to analyze differences in the dimensions of HIV risk between groups, configured on the basis of lifestyle quality. Post hoc analyses were implemented to examine specific differences between groups, with the Bonferroni adjustment for multiple comparisons (post hoc). These analyses allowed us to identify specific differences between three or more group means when an analysis of variance (MANOVA) F test was significant. Multiple linear regression analyses were conducted to identify significant personal and lifestyle predictors for each of the HIV risk analyzed factors. The chi-square statistic was employed to analyze differences in the proportion of individuals in high (under the mean in the sub-scales of the CPS) and low (above the mean in the sub-scales of the CPS) risk of HIV, depending on their higher (scores above the mean in the overall score of SLIQ; that is to say, ≥6) or lower (scores under the mean in the overall score of SLIQ; that is to say, ≤5) adherence to a total healthy lifestyle. Odds ratios of risk of HIV were also calculated. All statistical analyses were performed using the IBM SPSS, Statistics software for Windows, Version 25.0, considering *p* < 0.05 as significant.

## 3. Results

### 3.1. Scores in Each Dimension of HIV Risk of Infection

Table 2 shows the descriptive scores on evaluated HIV risk factors of the participants in the study and the percentage of participants at low and high risk in each HIV risk dimension.

### 3.2. Differences in Risk of HIV Infection Depending on Personal and Sociodemographic Characteristics

To evaluate differences in HIV risk dimensions based on personal and sociodemographic characteristics, participants were classified into two groups in each evaluated personal and sociodemographic factor (see Table 3).

In the case of K-HIV, differences were found by age [t(333) = −2.239, *p* = 0.026, *d* = 0.24] and employment status [t(333) = −3.365, *p* = 0.001, *d* = 0.36]. Those older and employed individuals exhibited higher knowledge about HIV.

For CUSEI, differences were found by age [t(333) = 2.230, *p* = 0.026, *d* = 0.24], relationship status [t(333) = 2.688, *p* = 0.008, *d* = 0.29], and employment status [t(333) = 2.038, *p* = 0.042, *d* = 0.22]. In this regard, those younger, single, and non-employed participants showed higher condom use intentions.

Regarding SAS-B, significant differences were found by age [t(333) = 2.205, *p* = 0.028, *d* = 0.24], relationship status [t(333) = 3.692, *p* = 0.0001, *d* = 0.40], and employment status [t(333) = 2.294, *p* = 0.022, *d* = 0.25]. Younger, single, and non-employed individuals exhibited higher safe sexual behaviors.

No significant differences were found in SEA, based on the personal characteristics of participants (Table 3).

### 3.3. Differences in Risk of HIV Infection Depending on Lifestyles

In order to identify possible differences in HIV risk dimensions based on lifestyle, participants were classified into three groups, based on their obtained scores in each of the lifestyle variables (see Table 4).

In the case of diet, differences between groups were found in CUSEI [F(2,332) = 4.622, *p* = 0.010, η^2^_partial_ = 0.027] and SAS-B [F(2,332) = 3.142, *p* = 0.045, η^2^_partial_ = 0.019]. In the case of CUSEI, post hoc analysis revealed differences between groups of good and moderate adherence to good diet, in comparison to the poor adherence diet group, the former exhibiting higher scores in CUSEI (*p* < 0.05). No significant differences were revealed in post hoc analyses for SAS-B (*p* > 0.05).

For exercise, differences between groups were found in SEA [F(2,332) = 6.776, *p* = 0.001, η^2^_partial_ = 0.039]. In this case, post hoc analysis indicated significant differences between participants who practiced vigorous exercise and moderate exercise, the former being at a lower risk in this dimension (*p* < 0.01).

With respect to alcohol consumption, significant differences between groups were found in CUSEI [F(2,332) = 6.090, *p* = 0.003, η^2^_partial_ = 0.035] and SAS-B [F(2,332) = 3.860, *p* = 0.022, η^2^_partial_ = 0.023]. After conducting the post hoc analysis, differences between groups were revealed in the cases of CUSEI and SAS-B, those individuals with moderate consumption being at higher risk in comparison to those participants who exhibited low consumption (*p* < 0.05).

In the case of smoking, differences were found for CUSEI [F(2,332) = 17.864, *p* = 0.0001, η^2^_partial_ = 0.097] and SAS-B [F(2,332) = 12.642, *p* = 0.0001, η^2^_partial_ = 0.071]. Post hoc analysis revealed differences between groups of current smokers and former smokers and non-smokers, showing lower scores in both dimensions the current smokers (*p* < 0.05). Similarly, former smokers showed lower scores than non-smokers (*p* < 0.05).

Regarding differences based on the total healthy lifestyle, differences were found in CUSEI [F(2,332) = 11.103, *p* = 0.0001, η^2^_partial_ = 0.063] and SAS-B [F(2,332) = 9.705, *p* = 0.0001, η^2^_partial_ = 0.055]. Post hoc analyses revealed differences between the three groups, showing higher scores in those participants with high adherence to a healthy lifestyle, in comparison to those with moderate and low adherence; and in those individuals with moderate adherence, in comparison to those with low adherence (*p* < 0.05; Table 4).

### 3.4. Personal and Lifestyles Predictors of HIV Risk Factors

Hierarchical regression analyses were performed to analyze the role of sociodemographic and lifestyles variables as predictors of HIV risk factors. Model 1 included only the evaluated sociodemographic characteristics of the sample. In Model 2, both sociodemographic and raw scores of lifestyle factors were included. All models for each HIV risk factor are included in Table 5 and Table 6.

### 3.5. Risk of HIV Infection Based on the Quality of Total Lifestyle

**Figure 1:**
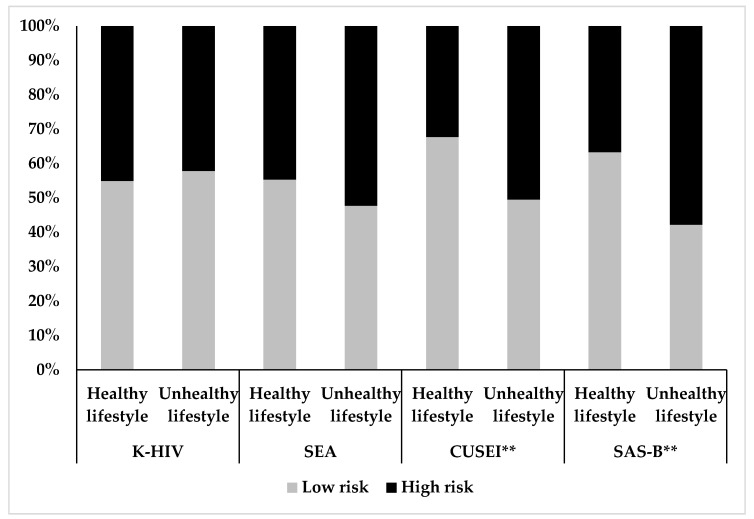
Risk of HIV infection based on the quality of total lifestyle. Knowledge about HIV (K-HIV), Self-Efficacy and Attitudes (SEA), Condom Use Intentions (CUSEI), Safe Sexual Behavior (SAS-B). Significant differences ** *p* < 0.01.

To evaluate the differences in the proportion of participants with high and low HIV risk depending on total lifestyle scores, differences between individuals with healthy and unhealthy lifestyle were analyzed (Figure 1). Differences were found only in CUSEI [Χ^2^ (1) = 10.269, *p* = 0.001] and SAS-B [Χ^2^ (1) = 13.280, *p* = 0.0001]. Regarding the HIV risk depending on the lifestyle, the odds ratio was calculated for each dimension: CUSEI [OR 2.135 (95% CI: 1.337, 3.408)] and SAS-B [OR 2.360 (95% CI: 1.480, 3.762)]. The obtained results indicate that individuals with an unhealthy lifestyle had twice the HIV risk in these sub-scales.

## 4. Discussion

The present study was aimed at the identification of the personal and lifestyle variables, which are related to HIV risk factors. To our knowledge, this is the first study to evaluate determinants of HIV risk in Spanish university students based on this broad approach. In this sense, the identification of personal and lifestyle correlates of HIV risk could allow clinicians and researchers to understand those most significantly related variables to the risk of HIV transmission in Spanish university students. This information will be very useful for the development of multicomponent prevention of HIV transmission strategies and, at the same time, the identification of those sub-groups of individuals at high HIV risk, which urgently need these types of prevention programs.

Based on the general scores obtained in the whole analyzed sample, the knowledge about HIV and its routes of transmission was adequate, although almost the half of the sample would need to improve its knowledge in this regard. Therefore, university students could likely benefit from more adequate and reliable information about HIV and its treatment. Regarding the use of condoms and safe sexual behavior, most of the participants obtained higher scores in this domain, demonstrating that it could be one of the main methods employed by participants for safe sexual intercourse.

As it can be observed, several personal characteristics seem to be related to various HIV risk factors, economic status being the only variable that did not show any association with HIV risk. Regarding lifestyle, most of the evaluated dimensions in this regard showed a relationship with some of the HIV risk variables: diet, smoking, and alcohol consumption being the lifestyle behaviors that exhibited associations with more HIV risk factors. Hence, when the risk of HIV was analyzed based upon the lifestyles exhibited by participants, those individuals with an unhealthy lifestyle were up to two times more likely to be at high risk for HIV transmission, in comparison to those who exhibited a healthy lifestyle (specifically, regarding the use of condoms and safe sexual behavior).

Regarding knowledge about HIV, only age and employment status were associated with this variable. Those participants over 18 years and those currently employed exhibited more knowledge about HIV. Taking into account that this dimension evaluates the quality and quantity of information that individuals have regarding routes of transmission, risky practices, preventive measures, or the impact of disease in people living with HIV, among others [42]; access to reliable and adequate information in this regard could be a significant protective factor for HIV transmission, as has been previously demonstrated in studies conducted with young people and people living with HIV [44,45,46]. It is likely that older individuals have had more opportunities to receive information about HIV and have developed more motivation and resources to access the information. It is also probable that older individuals have more active sexual relationships and are more interested in HIV education. Taking into account that no differences were found in this variable, in the case of lifestyle, access to HIV information and education received seems to be more influenced by personal variables than those regarding the lifestyles of individuals.

In the case of self-efficacy and attitudes towards the use of condoms—that is, the perceived ability of participants to use condoms, refuse unsafe sexual intercourse, and having knowledge about the negative influences of certain contextual factors (e.g., drugs)—significant differences were found between participants, depending of the intensity of physical activity. Those individuals who developed more intense physical exercise exhibited higher self-efficacy and positive attitudes regarding the use of condoms than those who developed less intense physical exercise. In this regard, although some contradictory results have been found in the previous literature, physical activity has been previously related to more safe sexual behaviors in adolescents [39]. It has been widely demonstrated that being physically active is a significant protective factor against the development of health risk behaviors in several populations, both young and adult individuals [47,48,49,50], such as drug consumption [47], alcohol consumption [48], smoking [49], unhealthy diet [50], or unsafe sexual behaviors [50], among others. Sports participation promotes the inclusion of individuals in prosocial and health environments, enhancing adaptive social relationships with peers and, hence, reducing the time of being involved in maladaptive contexts [50].

Moreover, as it has been pointed out by previous research, it seems that adolescents who practice vigorous exercise pay greater attention to their health status, avoiding any harmful behavior such as risky sexual behaviors [51]. This fact could explain the obtained results, as individuals engaged in vigorous exercise develop high self-efficacy towards the use of condoms, a healthy and protective measure, in order to maintain their positive health status and their physical performance [51]. In contrast, those university students who do not participate in vigorous physical activities may attribute less importance to healthy lifestyles and, hence, may exhibit lower self-efficacy in starting and maintaining healthy behaviors, becoming involved in risky sexual behaviors more frequently.

Differences in condom use intentions and safe sexual behavior were found for several personal dimensions and lifestyle variables. Younger, single, and non-employed participants exhibited higher intentions in the use of condoms and the development of safe sexual behavior. Further, those individuals who followed a good diet and consumed lower amounts of alcohol and tobacco also had higher intentions for condom use and safe sexual behavior performance. Generally, the obtained results were similar to the conclusions reported in previous research. A diminishing trend in the employment of condoms in sexual intercourse has been reported as the years go by [52]. Hence, it seems that the condom use declines with age [52]. Regarding relationship status, this variable is a stronger moderating factor, which modulates the impact of several psychological predictors on condom use in university students [53,54].

Generally, those individuals who are in a relationship use condoms less frequently than their single counterparts [54,55]. In this sense, in a longitudinal study in which 869 women attending two urban clinics for sexually transmitted diseases were evaluated, it was identified that condom use was modified by partner type, as its use decreases as the partner changes from new to regular [55]. Although several mechanisms have been proposed in the previous literature, one of the most important explanations could be based in the safety sensation that follows from being in a relationship. In this sense, a previous study conducted with university students has pointed out that being in a relationship entails a higher safety sensation and individuals do not perceive themselves to be a risk of being transmitted an STD [54]. Moreover, the use of a condom in a relationship could be perceived as a lack of trust in the partner [3], and individuals may avoid their employment in order to not generate couple problems.

Regarding lifestyle, although diet characteristics have not been previously analyzed in this sense, similar results have been obtained when food insecurity has been related to sexual behavior in adolescents and young adults [32,33,34]. Similarly, in the case of alcohol, various previous studies have found a significant positive association between alcohol consumption and HIV risk in young and adult populations [56,57,58]. Although smoking was associated, in classical research, with HIV risk [23,24,25,26,27], few studies have been conducted recently. In the case of alcohol, several neurocognitive explanations have been proposed, as authors identified an overlapping mechanism for the explanation of alcohol consumption and sexual risk behaviors in youth [59]. In this sense, deficits in neural areas directly related to decision-making could serve as the basis for the assumption of risky health behaviors. The influence of higher alcohol consumption in these areas could also affect the planification and decision of condom use, with youth having higher alcohol intake being more vulnerable to involvement in sexual risky behaviors. As has been proposed, these mechanisms could be extended to the explanation of the association between HIV risky behaviors and the rest of unhealthy lifestyle factors, as previous research has identified clusters of lifestyles and health risk behaviors, which share similar characteristics in college students [60]. In this regard, previous studies have identified an association between poor diet and tobacco consumption with negative cognitive effects, especially during youth [61,62,63]. Individuals with higher adherence to a poor diet and higher alcohol and tobacco consumption likely suffer from negative cognitive effects, such as deficits in decision-making and impulsiveness and, hence, become involved in risky sexual behaviors more frequently [64]. Beyond the cognitive mechanisms, as it has been previously proposed with respect to physical exercise, people who generally follow a healthy lifestyle are more worried about their health status, hence developing several healthy behaviors incompatible with risky sexual practices.

Although the present study entails a significant advance in the comprehension of the association between personal and lifestyle characteristics of the university students living in Spain with HIV transmission risk, some limitations should be addressed. The cross-sectional and descriptive characteristics of the study did not allow us to establish causality in the obtained results. Longitudinal methodologies could entail an advance in this regard and would allow for the identification of causal relationships between lifestyle factors and HIV risky behaviors. Furthermore, lifestyles were evaluated with a brief self-reported questionnaire, and data reported by this type of measures could be influenced by desirability and recall bias. Moreover, the small sample size and the fact that it was recruited following a convenience sampling method could limit the generalization ability of the obtained results. Future studies should be developed with larger, randomized, and more representative samples. In any case, the present study evaluated the associations between several personal and lifestyle characteristics of university students and multiple domains of HIV risk, thus being one of the few studies to analyze them in such a comprehensive mode.

## 5. Conclusions

The obtained results identify the personal and lifestyles factors associated with a higher risk for HIV transmission in Spanish university students. To our knowledge, this is one of the few studies that has evaluated the associations of various personal and lifestyle characteristics and several HIV risk factors in this population. As has been previously indicated, different relationships were found between the analyzed factors and HIV risk dimensions, from which it can be derived that personal and lifestyle factors could serve either as risk or protective factors, and that specific HIV risk dimensions should be taken into account. Being older, employed, and having a stable relationship seems to be related with higher HIV risk. Moreover, a poor diet, a lower intensity of physical exercise, higher alcohol consumption, and smoking are significantly associated with more HIV risk. This information could be beneficial for the development of HIV preventive programs in Spanish university students, mostly oriented to the promotion of healthy lifestyles, such as healthy diet or physical exercise, and alcohol and tobacco consumption reduction. Future studies should analyze, in a more comprehensive manner, how specific lifestyle factors are related to different HIV risk dimensions, in order to provide useful information on how the characteristics of each of the evaluated lifestyles moderate the risk of HIV transmission. In this sense, further research to explore correlations or even causal relationships between specific and smaller personal and lifestyle variables and HIV risk of transmission in this population is required. Although several personal and lifestyle variables have been taken into account, other social and cultural factors that were not evaluated in the present research could have significant impacts on HIV risk in this population. Future studies analyzing the sociocultural dimensions on risk of HIV transmission in this population are required.

## Figures and Tables

**Table 1:** Sociodemographic and lifestyle characteristics of the sample.

Variable	Categories	Total Sample N = 335
Age		19.62 ± 2.22
Sex	Male	71 (21.2%)
	Female	264 (78.8%)
Relationship status	Single	234 (69.9%)
	In a relationship	101 (30.1%)
Employment status	Employed	52 (15.5%)
	Non-employed	283 (84.5%)
Economic status ^a^	EUR < 500	284 (84.8%)
	EUR > 500	51 (15.2%)
Sexual orientation	Heterosexual	250 (74.6%)
	Non-heterosexual	85 (25.4%)
Diet	Poor	123 (36.7%)
	Moderate	162 (48.4%)
	Good	50 (14.9%)
Exercise	Light	49 (14.6%)
	Moderate	101 (30.1%)
	Vigorous	185 (55.2%)
Alcohol consumption	Low	273 (81.5%)
	Moderate	42 (12.5%)
	High	20 (6%)
Smoking	Non-smoker	137 (40.9%)
	Former smoker	147 (43.9%)
	Current smoker	51 (15.2%)
Life stress	Low	58 (17.3%)
	Moderate	191 (57%)
	High	86 (25.7%)
Total Healthy Lifestyle	Low	109 (32.5%)
	Moderate	151 (45.1%)
	High	75 (22.4%)

^a^ Average monthly economic income during the last year.

**Table 2:** Scores in dimensions of risk of HIV infection in the total sample.

Variable	Min.	Max.	Mean	SD	N (%)	
					Low Risk	High Risk
K-HIV	7.00	24.00	17.74	2.83	187 (55.8%)	148 (44.2%)
SEA	15.00	45.00	29.49	4.44	177 (52.8%)	158 (47.2%)
CUSEI	1.00	18.00	11.87	3.89	207 (61.8%)	128 (38.2%)
SAS-B	0.00	18.00	11.33	4.33	189 (56.4%)	146 (43.6%)

Knowledge about HIV (K-HIV), Self-Efficacy and Attitudes (SEA), Condom Use Intentions (CUSEI), Safe Sexual Behavior (SAS-B).

**Table 3:** Differences in risk of HIV infection between participants based on personal characteristics.

Variable	Categories	K-HIV	SEA	CUSEI	SAS-B
Age	18 years (n = 163)	**17.39 ± 2.88**	29.22 ± 4.89	**12.35 ± 3.70**	**11.87 ± 4.11**
	>18 years (n = 172)	**18.08 ± 2.74**	29.75 ± 4.06	**11.41 ± 4.01**	**10.83 ± 4.49**
Sex	Female (n = 264)	17.85 ± 2.80	29.34 ± 4.53	11.81 ± 3.98	11.19 ± 4.42
	Male (n = 71)	17.33 ± 2.89	30.07 ± 4.05	12.09 ± 3.51	11.85 ± 3.98
Relationship status	Single (n = 234)	17.76 ± 2.89	29.33 ± 4.67	**12.24 ± 3.94**	**11.90 ± 4.23**
	In a relationship (n = 101)	17.70 ± 2.69	29.87 ± 3.85	**11.00 ± 3.64**	**10.02 ± 4.31**
Employment status	Employed (n = 52)	**18.94 ± 2.54**	29.57 ± 5.01	**10.86 ± 3.96**	**10.07 ± 4.37**
	Non-employed (n = 283)	**17.52 ± 2.83**	29.48 ± 4.33	**12.05 ± 3.85**	**11.56 ± 4.29**
Economic status ^a^	<500 euros (n = 284)	17.68 ± 2.79	29.48 ± 4.37	11.83 ± 3.86	11.41 ± 4.22
	>500 euros (n = 51)	18.07 ± 3.02	29.56 ± 4.83	11.82 ± 4.09	10.88 ± 4.95
Sexual Orientation	Heterosexual (n = 250)	17.71 ± 2.91	29.44 ± 4.46	11.71 ± 4.06	11.21 ± 4.33
	Non-heterosexual (n = 85)	17.84 ± 2.59	29.65 ± 4.40	12.34 ± 3.29	11.70 ± 4.36

Knowledge about HIV (K-HIV), Self-Efficacy and Attitudes (SEA), Condom Use Intentions (CUSEI), Safe Sexual Behavior (SAS-B). Significant differences (*p* < 0.05) between groups (groups without significant differences are not indicated) calculated with the T-test are marked in bold. ^a^ Average monthly economic income during the last year.

**Table 4:** Differences in the main dimensions of risk of HIV infection between participants based on lifestyles.

Variable	Categories	K-HIV	SEA	CUSEI	SAS-B
Diet	Poor (n = 123)	17.43 ± 2,84	28,80 ± 4.74	**11.04 ± 4.49**	**10.60 ± 4.95**
	Moderate (n = 162)	17.83 ± 2.83	29.75 ± 4.17	**12.29 ± 3.50**	**11.61 ± 3.93**
	Good (n = 50)	18.22 ± 2.75	30.34 ± 4.36	**12.56 ± 3.14**	**12.20 ± 3.72**
Exercise	Light (n = 49)	17.64 ± 2.72	**29.14 ± 3.91**	10.89 ± 4.70	10.20 ± 5.33
	Moderate (n = 101)	17.37 ± 2.87	**28.28 ± 4.86**	11.67 ± 3.93	11.09 ± 4.30
	Vigorous (n = 185)	18.02 ± 2.82	**30.24 ± 4.18**	12.23 ± 3.59	11.76 ± 4.01
Alcohol consumption	Low (n = 273)	17.84 ± 2.93	29.61 ± 4.42	**12.19 ± 3.87**	**11.63 ± 4.33**
	Moderate (n = 42)	17.19 ± 2.32	28.40 ± 4.91	**10.02 ± 3.68**	**9.73 ± 4.09**
	High (n = 20)	17.60 ± 2.25	30.15 ± 3.39	**11.30 ± 3.59**	**10.60 ± 4.28**
Smoking	Non-smoker (n = 137)	17.62 ± 2.79	29.18 ± 4.38	**13.07 ± 3.63**	**12.50 ± 4.20**
	Former smoker (n = 147)	17.74 ± 2.94	29.49 ± 4.50	**11.56 ± 3.54**	**11.00 ± 4.08**
	Current smoker (n = 51)	18,09 ± 2.58	30,33 ± 4.38	**9.52 ± 4.33**	**9.15 ± 4.48**
Life stress	Low (n = 58)	17.17 ± 2.88	29.43 ± 3.81	11.74 ± 3.82	11.51 ± 4.37
	Moderate (n = 191)	17.75 ± 2.65	29.58 ± 4.59	11.96 ± 3.80	11.57 ± 4.21
	High (n = 86)	18,10 ± 3,12	29.34 ± 4,53	11.75 ± 4,15	10,68 ± 4.56
	Low (n = 109)	17.33 ± 2.76	28.93 ± 4.72	**10.55 ± 4.26**	**9.96 ± 4.67**
Total Healthy Lifestyle	Moderate (n = 151)	17.66 ± 2.90	29.63 ± 4.31	**12.21 ± 3.60**	**11.69 ± 4.08**
	High (n = 75)	17.93 ± 2.81	30.02 ± 4.25	**13.09 ± 3.32**	**12.61 ± 3.81**

Knowledge about HIV (K-HIV), Self-Efficacy and Attitudes (SEA), Condom Use Intentions (CUSEI), Safe Sexual Behavior (SAS-B). Significant differences (*p* < 0.05) between groups (groups without significant differences are not indicated) calculated with the ANOVA test are marked in bold.

**Table 5:** Predictive value of personal and lifestyles variables on K-HIV and SEA dimensions of HIV risk factors.

Variable	Knowledge about HIV (K-HIV)					Variable	Attitudes and Perceived Self-Efficacy (SEA)				
	**Model 1**						**Model 1**				
	**B**	**SE**	**β**	***p***	**95% CI**		**B**	**SE**	**β**	***p***	**95% CI**
Age	0.073	0.075	0.057	0.334	[−0.075, 0.221]	Age	0.188	0.119	0.094	0.117	[−0.047, 0.423]
Sex	0.391	0.381	0.056	0.306	[−0.358, 1.139]	Sex	−0.910	0.604	−0.084	0.133	[−2.098, 0.279]
Relationship status	−0.076	0.338	−0.012	0.822	[−0.740, 0.588]	Relationship status	0.506	0.536	0.052	0.346	[−0.548, 1.560]
Employment status	1.230	0.460	0.158	0.008	[0.326, 2.135]	Employment status	−0.150	0.730	−0.012	0.837	[−1.586, 1.285]
Economic status	−0.019	0.449	−0.002	0.966	[−0.902, 0.864]	Economic status	−0.203	0.712	−0.016	0.776	[−1.604, 1.199]
Sexual orientation	−0.017	0.357	−0.003	0.962	[−0.719, 0.685]	Sexual orientation	0.249	0.566	0.024	0.660	[−0.865, 1.363]
F(6334) = 2.240, *p* = 0.039 R^2^ = 0.022						F(6334) = 0.934, *p* = 0.471 R^2^ = −0.001					
	**Model 2**						**Model 2**				
	**B**	**SE**	**β**	***p***	**95% CI**		**B**	**SE**	**β**	***p***	**95% CI**
Age	0.045	0.078	0.035	0.564	[−0.108, 0.198]	Age	0.195	0.123	0.098	0.112	[−0.046, 0.437]
Sex	0.412	0.395	0.060	0.297	[−0.365, 1.189]	Sex	−0.792	0.622	−0.073	0.204	[−2.015, 0.432]
Relationship status	−0.077	0.337	−0.012	0.820	[−0.740, 0.586]	Relationship status	0.606	0.531	0.063	0.254	[−0.438, 1.650]
Employment status	1.259	0.459	0.161	0.006	[0.357, 2.161]	Employment status	−0.211	0.722	−0.017	0.770	[−1.631, 1.209]
Economic status	0.003	0.446	0.000	0.995	[−0.874, 0.880]	Economic status	−0.108	0.702	−0.009	0.878	[−1.488, 1.273]
Sexual orientation	−0.052	0.359	−0.008	0.885	[−0.758, 0.654]	Sexual orientation	0.189	0.565	0.019	0.738	[−0.922, 1.300]
Diet	0.032	0.052	0.036	0.537	[−0.070, 0.133]	Diet	0.160	0.081	0.115	0.049	[0.001, 0.320]
Exercise	0.073	0.032	0.135	0.023	[0.010, 0.136]	Exercise	0.112	0.050	0.132	0.026	[0.013, 0.212]
Alcohol consumption	−0.057	0.036	−0.098	0.110	[−0.127, 0.013]	Alcohol consumption	−0.021	0.056	−0.022	0.715	[−0.131, 0.090]
Smoking	−0.372	0.246	−0.093	0.132	[−0.857, 0.113]	Smoking	−0.748	0.388	−0.119	0.055	[−1.511, 0.015]
Life stress	−0.086	0.138	−0.036	0.535	[−0.356, 0.185]	Life stress	0.037	0.217	0.010	0.866	[−0.389, 0.463]
F(11,334) = 2.171, *p* = 0.016 R^2^ = 0.037 ΔR^2^ = 0.029, *p* = 0.072						F(11,334) = 1.977, *p* = 0.030 R^2^ = 0.031 ΔR^2^ = 0.046, *p* = 0.008					

**Table 6:** Predictive value of personal and lifestyle variables on CUSEI and SAS-B dimensions of HIV risk factors.

Variable	Condom Use Intention (CUSEI)					Variable	Safe Sexual Behavior (SAS-B)				
	**Model 1**						**Model 1**				
	**B**	**SE**	**β**	***p***	**95% CI**		**B**	**SE**	**β**	***p***	**95% CI**
Age	−0.155	0.103	−0.089	0.133	[−0.358, 0.048]	Age	−0.204	0.113	−0.105	0.073	[−0.427, 0.019]
Sex	−0.088	0.521	−0.009	0.866	[−1.113, 0.936]	Sex	−0.377	0.573	−0.036	0.512	[−1.505, 0.751]
Relationship status	−1.165	0.462	−0.138	0.012	[−2.074, −0.257]	Relationship status	−1.770	0.509	−0.188	0.001	[−2.771, −0.770]
Employment status	−1.083	0.629	−0.101	0.086	[−2.321, 0.155]	Employment status	−1.189	0.693	−0.099	0.087	[−2.552, 0.174]
Economic status	0.354	0.614	0.033	0.565	[−0.854, 1.562]	Economic status	−0.031	0.676	−0.003	0.963	[−1.361, 1.299]
Sexual orientation	0.710	0.488	0.080	0.147	[−0.250, 1.670]	Sexual orientation	0.641	0.537	0.064	0.234	[−0.416, 1.698]
F(6334) = 2.742, *p* = 0.013 R^2^ = 0.030						F(6334) = 4.203, *p* = 0.000 R^2^ = 0.054					
	**Model 2**						**Model 2**				
	**B**	**SE**	**β**	***p***	**95% CI**		**B**	**SE**	**β**	***p***	**95% CI**
Age	−0.168	0.101	−0.096	0.098	[−0.367, 0.031]	Age	−0.197	0.114	−0.101	0.084	[−0.420, 0.027]
Sex	0.206	0.514	0.022	0.688	[−0.805, 1.217]	Sex	−0.074	0.576	−0.007	0.898	[−1.207, 1.059]
Relationship status	−1.156	0.438	−0.137	0.009	[−2.019, −0.294]	Relationship status	−1.751	0.492	−0.185	0.000	[−2.718, −0.784]
Employment status	−0.859	0.596	−0.080	0.151	[−2.032, 0.314]	Employment status	−0.971	0.669	−0.081	0.147	[−2.287, 0.344]
Economic status	0.374	0.580	0.035	0.519	[−0.767, 1.515]	Economic status	0.011	0.650	0.001	0.986	[−1.268, 1.290]
Sexual orientation	0.975	0.467	0.109	0.038	[0.057, 1.893]	Sexual orientation	0.902	0.523	0.091	0.086	[−0.128, 1.931]
Diet	0.077	0.067	0.063	0.254	[−0.055, 0.209]	Diet	0.114	0.075	0.083	0.131	[−0.034, 0.262]
Exercise	0.062	0.042	0.084	0.136	[−0.020, 0.144]	Exercise	0.045	0.047	0.054	0.333	[−0.047, 0.137]
Alcohol consumption	−0.090	0.046	−0.112	0.053	[−0.181, 0.001]	Alcohol consumption	−0.088	0.052	−0.098	0.092	[−0.190, 0.014]
Smoking	1.384	0.320	0.251	0.000	[0.754, 2.014]	Smoking	1.269	0.359	0.206	0.000	[0.562, 1.976]
Life stress	−0.052	0.179	−0.016	0.771	[−0.404, 0.300]	Life stress	0.096	0.201	0.026	0.633	[−0.299, 0.491]
F(11,334) = 5.849, *p* = 0.013 R^2^ = 0.138 ΔR^2^ = 0.118, *p* = 0.000						F(11,334) = 5.455, *p* = 0.000 R^2^ = 0.128 ΔR^2^ = 0.085, *p* = 0.000					
